# Differential Expression and Cell-Type Specificity of Perineuronal Nets in Hippocampus, Medial Entorhinal Cortex, and Visual Cortex Examined in the Rat and Mouse

**DOI:** 10.1523/ENEURO.0379-16.2017

**Published:** 2017-06-07

**Authors:** Kristian Kinden Lensjø, Ane Charlotte Christensen, Simen Tennøe, Marianne Fyhn, Torkel Hafting

**Affiliations:** 1 Department of Biosciences, University of Oslo, Oslo, 0317, Norway; 2 Institute of Basic Medical Sciences, University of Oslo, Oslo, 0317, Norway; 3 Department of Informatics, University of Oslo, Oslo, 0317, Norway; 4 Center for Integrative Neuroplasticity, University of Oslo, Oslo, 0317, Norway

**Keywords:** CA2, entorhinal, hippocampus, parvalbumin, WFA

## Abstract

Perineuronal nets (PNNs) are specialized extracellular matrix (ECM) structures that condense around the soma and proximal dendrites of subpopulations of neurons. Emerging evidence suggests that they are involved in regulating brain plasticity. However, the expression of PNNs varies between and within brain areas. A lack of quantitative studies describing the distribution and cell-specificity of PNNs makes it difficult to reveal the functional roles of PNNs. In the current study, we examine the distribution of PNNs and the identity of PNN-enwrapped neurons in three brain areas with different cognitive functions: the dorsal hippocampus, medial entorhinal cortex (mEC) and primary visual cortex (V1). We compared rats and mice as knowledge from these species are often intermingled. The most abundant expression of PNNs was found in the mEC and V1, while dorsal hippocampus showed strikingly low levels of PNNs, apart from dense expression in the CA2 region. In hippocampus we also found apparent species differences in expression of PNNs. While we confirm that the PNNs enwrap parvalbumin-expressing (PV+) neurons in V1, we found that they mainly colocalize with excitatory CamKII-expressing neurons in CA2. In mEC, we demonstrate that in addition to PV+ cells, the PNNs colocalize with reelin-expressing stellate cells. We also show that the maturation of PNNs in mEC coincides with the formation of grid cell pattern, while PV+ cells, unlike in other cortical areas, are present from early postnatal development. Finally, we demonstrate considerable effects on the number of PSD-95-gephyrin puncta after enzymatic removal of PNNs.

## Visual Abstract

**Figure:**
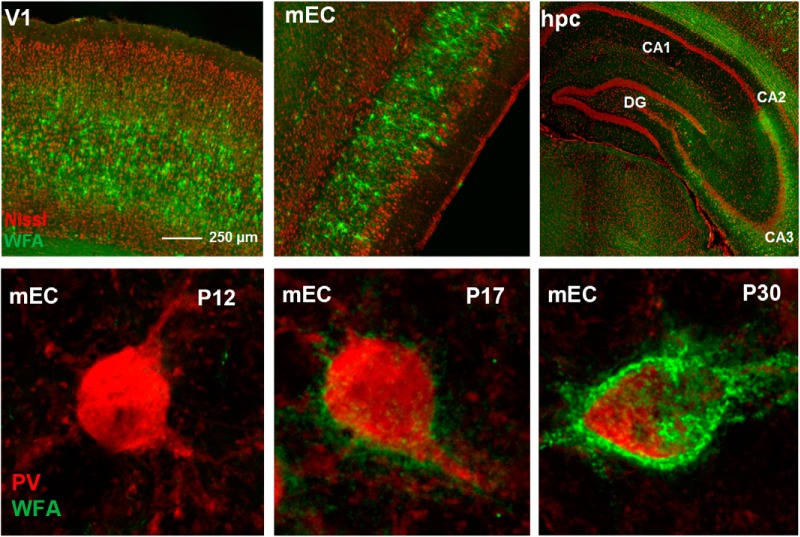

## Significance Statement

Perineuronal nets (PNNs) are proposed as a key regulator of plasticity in the adult brain. However, PNNs are not uniformly expressed and their prevalence and cell-type specificity is lacking for several well-studied brain regions. Here, we examine the visual cortex, hippocampus and the medial entorhinal cortex (mEC) and show that the density and structure of PNNs as well their cell-type specificity are different between brain areas and between rats and mice. We show for the first time a dense expression of PNNs in mEC and that their assembly during postnatal development coincides with reports on development of the grid cell’s activity pattern. The variable expression patterns of PNNs may reflect different levels of plasticity and wiring of the neural networks.

## Introduction

Perineuronal nets (PNNs) are specialized structures of extracellular matrix (ECM) that condense around the cell soma and proximal dendrites of subpopulations of neurons (Hockfield and McKay, 1983; Celio and Blumcke, 1994). Recent work indicates that PNNs play a role in regulating plasticity during development, learning, and memory processing (Bartus et al., 2012). Moreover, dysfunctional regulation of PNNs may be linked to impaired synaptic function in some psychiatric disorders such as schizophrenia (Pantazopoulos and Berretta, 2016).

The PNNs are found throughout the brain with profound variations in expression pattern and morphology between brain regions (Seeger et al., 1994). Their main components are hyaluronic acid, link proteins, tenascins, and chondroitin sulfated proteoglycans (Deepa et al., 2006; Giamanco and Matthews, 2012), that are heterogeneously expressed causing differences in the PNN structures (Seeger et al., 1994; Lander et al., 1997; Wegner et al., 2003; Deepa et al., 2006; Dauth et al., 2016). The PNNs mainly enwrap a subpopulation of inhibitory neurons, the fast-spiking parvalbumin-expressing (PV+) inhibitory neurons, but the overlap is not exclusive (Kosaka and Heizmann, 1989; Härtig et al., 1992; Wegner et al., 2003; Alpar et al., 2006). In the neocortex, the PV+ cells mature in parallel with the assembly of the PNNs late in postnatal development as the critical period comes to an end (Hockfield et al., 1990; Pizzorusso et al., 2002; Ye and Miao, 2013). The PV+ cells have been proposed as key regulators of plasticity, both during development and in adulthood (Hensch, 2005; Donato et al., 2013). The PNNs may support the high activity of PV+ cells through ion buffering, protection against reactive oxygen radicals and their role in plasticity through capture of the transcription factor Otx2 (Beurdeley et al., 2012; Cabungcal et al., 2013; Liu et al., 2013; Morawski et al., 2015). Thus, PNNs may restrict plasticity either indirectly through their effect on PV+ cells or directly by acting as a structural barrier inhibiting synapse remodeling and axonal sprouting. Recent work has demonstrated that PNN removal reduces excitability and spiking activity of putative PV+ neurons (Balmer, 2016; Lensjø et al., 2017). Removing the PNNs enzymatically in adult animals increases plasticity in several brain areas, including the visual (Pizzorusso et al., 2002) and auditory cortices (Happel et al., 2014), perirhinal cortex (Romberg et al., 2013), hippocampus (Hylin et al., 2013), and amygdala (Gogolla et al., 2009).Taken together, this suggests that the function of PNNs is tightly linked to PV+ cell function. However, PNNs enwrapping other cell types have been found in several brain areas (Alpar et al., 2006; Carstens et al., 2016; Morikawa et al., 2017). Furthermore, it remains unclear if the level of PNN expression in a brain area is correlated with the level of plasticity in that area.

Despite increased attention to PNNs recently, quantification of PNN expression and their cell-type specificity is limited. We have therefore examined PNNs in three areas of the rodent brain with known different functions: the dorsal hippocampus, medial entorhinal cortex (mEC) and primary visual cortex (V1). The hippocampus is critical for encoding new memories and is reciprocally connected with mEC. The mEC is believed to be the hub in a distributed neural network coding for spatial representation that is fed into the hippocampus and used as a critical component of episodic memories (Buzsaki and Moser, 2013). The V1 has been a canonical system to study sensory processing and critical period plasticity for decades (Hensch, 2005; Wandell and Smirnakis, 2009). While PNNs have been investigated in detail in the V1, both in adults and during postnatal development, it remains elusive how the findings from this primary sensory cortex correspond to the development and expression pattern in mEC.

We show that the expression of PNNs is highly variable within and between the hippocampus, V1, and mEC. We have identified several distinct cell types associated with PNNs, large differences in PNN expression between mice and rats, and considerable but variable effects on the density of PSD-95 and gephyrin-positive puncta after enzymatic removal of PNNs in V1 and mEC in rats. Our results demonstrate that PNNs are heterogeneous in expression, cell-type specificity and morphology. This variability may point to different roles of the PNNs perhaps reflecting different levels of plasticity or wiring of the neural networks.

## Materials and Methods

### Animals

Fourteen adult (three to five months old) and seven juvenile (postnatal day 10 (P10) to P30) male Long Evans rats and five male c57/bl6 mice (three to five months old) were used for this study. The animals were locally bred and maintained at the animal facility at the Department of Biosciences, University of Oslo. The animals were housed two to three (rats) or five to eight (mice) together, with a 12/12 h light/dark cycle and food and water *ad libitum*. All animal procedures were approved by the Norwegian Animal Research Committee before initiation.

### Brain sectioning

The animals were anesthetized in an induction chamber with isoflurane mixed with air. They were then deeply anesthetized with an intraperitoneal injection of sodium pentobarbital (50 mg/kg) and intracardially perfused with 0.9% NaCl followed by 4% paraformaldehyde (PFA) in 1× PBS. The brains were dissected out and postfixed in 4% PFA overnight (except for staining synaptic markers PSD-95 and gephyrin, as well as for tissue injected with retrograde tracer; Table 1). The tissue was then cryoprotected in a solution of 30% sucrose in 1× PBS for 3 d at 4°C, flash frozen, and sectioned with a cryostat in 40-µm-thick sections. The sections were collected from the cryostat with a fine brush and placed in 1× PBS.

**Table 1:** Overview of antibodies used

Primary antibodies used						
Primary antibody	Reagent	Dilution	Provided by	RRID	Postfix	Other
WFA	N-acetylgalactosamine	1:200	Sigma	AB_2620171	ON	
Rabbit anti-PV	Parvalbumin	1:2000	Swant	AB_10000344	ON	
						
Rabbit anti-CamKII	CamKII α-subunit	1:500	Abcam	AB_447192	ON
Rabbit antigephyrin	Gephyrin	1:1000	Abcam	AB_2112628	2h	
						0.05% Tween 20
Mouse anti-PSD-95	PSD-95	1:500	Abcam	AB_303248	2h	
						
Mouse anti-CS-6	Chondroitin 6-sulfate stubs	1:1000	Millipore	AB_11214309	ON	
after chABC digestion
Mouse antireelin	Reelin	1:500	Abcam	AB_1603148	ON	
Rabbit anti-GFAP	Glial fibrillary acidic protein	1:500	Dako	AB_10013382	ON	
						
Rabbit anticalbindin	Calbindin	1:5000	Swant	AB_10000340	ON	
					
Neurotrace Nissl	Nissl bodies	1:100	Dako	AB_10013382	ON
					
Rabbit anti-PCP4	Purkinje cell protein 4	1:200	Sigma	AB_1669533	ON

### Immunohistochemistry

Staining was performed on free-floating sections with constant agitation. Sections were initially blocked with a solution containing 1-2% bovine serum albumin and 0.03% Triton X-100 for 1 h and incubated with the primary antibody overnight (primary antibodies are listed in Table 1). Sections were rinsed 3 × 5 min in 1× PBS and incubated with secondary antibody for 1-4 h. After being washed in 1× PBS, sections were mounted with FluorSave Reagent (Merck Millipore).

### Microscopy

Overview images were acquired through an Axioplan 2 microscope (Carl Zeiss) with a 10× objective, and high-resolution images were stitched together using the MosaiX extension in the AxioVision software (Carl Zeiss).

Detailed images of PNNs, synaptic markers and colocalization analysis were acquired using a 20× or 60× PlanApo objective on a FluoView FV 1000 confocal microscope (Olympus) using the FV1000 software (version 1.7a). Images were acquired in a stepwise manner through the z-plane, each step separated by 1.4 µm (20× objective) or 0.45 µm (60× objective).

### Image analysis

Image analysis was performed using Adobe Photoshop CS4 (Adobe Systems) and ImageJ (NIH). Analysis of puncta and 3D visualization was performed in Imaris (Bitplane).

As a general note for quantification of the number of PNNs or number of cells, and their colocalization, we attempted an automated approach using ImageJ and by manual counting. Both yielded similar results, but the automatic approach required the removal of background before analysis, which resulted in many false negatives when compared with the raw data. Without background subtraction, the automated approach gave rise to a substantial number of false positives. We therefore chose to perform all colocalization analysis by manual counting, by overlaying the sections with a grid and using the counter tool. All images were counted by two investigators independently. To investigate the fraction of PNN-enwrapped cells we used sections stained with *Wisteria floribunda* agglutinin (WFA) and fluorescent Nissl. In the hippocampus, the cells were too densely packed to be able to separate them and get an accurate cell number; this region therefore was left out of the analysis.

For analysis of both number and distribution of PSD-95 and gephyrin puncta, we always compared the treated and untreated hemispheres from the same rat and normalized values to the mean of the same area in the control hemisphere. This was important to account for variability between animals and staining quality. The control measurements from chondroitinase ABC (chABC)-treated rats were not different from measurements in control or treated sham animals. The sections from each rat were treated in an identical fashion, and stained and imaged in parallel. We used the mean from two to three sites in each of two sections per hemisphere to determine the number of puncta. Analysis of the number of PSD-95 and gephyrin puncta was conducted using the built-in spot detection algorithm in Imaris (γ 1.79, quality 80). The coordinates of the puncta were then exported for further analysis of position and clustering using custom written Python code.

### Clustering analysis

The data from the imaging of gephyrin and PSD-95 puncta were clustered with the friends-of-friends (FOF) algorithm described by Davis et al. (1985). The FOF algorithm has one free parameter, the linking length between two points. Any two puncta that lies closer than this length are linked together. A cluster is then all puncta that are connected to each other through a network of linked puncta. Measurements of the distance between a selected set of puncta showed that 2 μm was a good choice of linking length, as a shorter or longer distance would yield clusters of very few puncta or only a few clusters with almost all puncta clustered, respectively. As the objective was to perform a comparison between the two datasets (chABC treated vs control), the exact choice of linking length is not important as long as it is kept constant.

### Injections of chABC, artificial cerebrospinal fluid (aCSF), and retrograde tracer

chABC from *Proteus vulgaris* (Amsbio) was diluted in filtered 1× PBS to an initial concentration of 61 U/mL, and stored at -20°C in smaller aliquots before surgery. Anesthesia was induced by placing the animals in an induction chamber with 5% isoflurane concentration. Animals were then placed in a stereotaxic frame and provided with isoflurane mixed with air at a constant flow of 2 l/min, through an anesthesia mask. They were given subcutaneous injections of buprenorphine (Temgesic, 0.04 mg/kg) and local subcutaneous injections of bupivacaine/adrenaline (Marcain adrenaline, 13.2 mg/kg) in the skin of the scalp before surgery began. The scalp was shaved and cleaned with 70% ethanol and chlorhexidine, and a small incision was made in the skin. Small craniotomies were made with a hand held dental drill. The microinjector (NanoJect II, Drummond Scientific) was mounted onto the stereotaxic frame and a glass pipette was filled with chABC mixed with fast green FCF (Sigma-Aldrich Chemie) to a final concentration of 48 U/ml, or aCSF (Harvard Apparatus) as a sham injection. A total of four unilateral injection sites were used for MEC. Stereotaxic coordinates were 0.5 mm anterior of the transverse sinus, 4.5 and 4.7 mm lateral of the midline, and 3.0 and 2.5 mm below dura mater with the pipette positioned at 15° angle in the sagittal plane and the tip pointing in the anterior direction. For V1 injections, we used three coordinates, all relative to lambda: 0.25 mm posterior and 4.5 mm lateral, 0.25 mm posterior and 4.9 mm lateral, and 0.75 mm posterior and 4.7 mm lateral. All the V1 injections were made at a depth of 0.6 mm depth, relative to the dura mater.

Injections at each site were performed in steps of 23 nl each, with a total volume of 368 nl for each position. The pipette was kept in place for 1-2 min to increase diffusion of chABC before the wound was cleaned and sutured shut. Animals were given subcutaneous injections of carprofen (Rimadyl, 5 mg/kg) and local anesthetic ointment (Lidocain) was applied. This was repeated for 3 d. Animals were sacrificed 7 d after surgery.

To perform retrograde labeling of neurons projecting from the mEC to the hippocampus, we used cholera toxin subunit B, conjugated to Alexa Fluor 594 (C22842, Life Technologies), diluted in 1× PBS (10% wt/vol). The procedure was conducted as described above. To target the projections from Layer II of mEC to dentate gyrus (DG) we used the following coordinates relative to bregma: 4.1 mm posterior, 2.6 mm lateral, and 3.5 mm below dura. While for injections in the CA1 aiming for projections from Layer III of mEC were 4.1 mm posterior, 2.6 mm lateral, and 2.1 mm below dura. We injected a total of 0.2 µl at each site over a period of 10 min. Medication procedures were identical to those described above. The animals were sacrificed after 5 d.

### Statistical analysis

All statistical analysis was performed using GraphPad Prism 6 (GraphPad) or SigmaPlot 12.0 (Systat Software). All data were tested for normality by Wilks-Shapiro test, and further analysis was performed accordingly (Student’s *t* test or Mann–Whitney *U* test).

## Results

**Figure 1.**
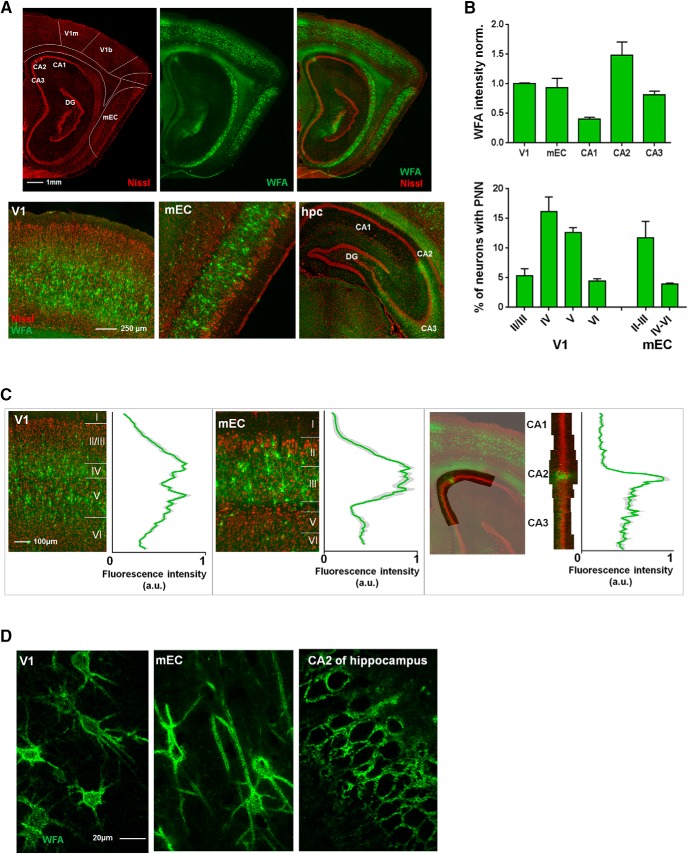
Laminar distribution and structural differences of WFA-positive PNNs in V1, mEC, and hippocampus. ***A***, Sagittal section of a rat brain stained with Neurotrace (Nissl bodies) and WFA. Brain areas are indicated based on the Paxinos atlas of the rat brain, with the areas of interest denoted. Lower panel shows the areas of interest highlighted, mEC in a sagittal plane, while V1 and hippocampus are shown in coronal plane. ***B***, Expression of PNNs was quantified by intensity measurement and counting the fraction of PNN-enwrapped cells. The intensity measurements showed that expression was highest in V1 and CA2. WFA intensity from all areas was normalized to the values from V1. The fraction of neurons enwrapped by PNNs were quantified by manual counting and showed a layer-specific expression of PNNs. While the area averages (8.4% for V1, 7.9% for mEC) of PNN-positive neurons were similar between the areas, the layer-specific expression was different. Measurements were performed in three sections from three rats, bar graphs shows mean ± SEM. ***C***, In V1, PNNs are uniformly expressed across cell layers, with the highest expression in Layer IV and the lower part of Layer V (left). In mEC, PNNs are densely expressed in Layer II/III with only sparse labeling in Layer V and VI (middle). The dorsal hippocampus (right) is largely void of PNNs, with the exception of area CA2 and partially CA3, which shows dense labeling of diffuse PNN-like structures. Intensity measurements across cell layers in V1 and mEC was performed in three sections from three rats and is shown as mean ± SEM. ***D***, The structure of WFA-positive PNNs is highly variable between brain areas. High-resolution images from the V1 (left) show PNNs enwrapping the soma and the most proximal dendritic segments; from the mEC (middle) where the PNNs enwrap the cell soma extending far out in the proximal dendrite, and from the CA2 (right) where the PNNs are diffuse in structure and only embody the cell soma and neuropil.

To investigate the expression of PNNs, we stained brain sections with WFA in the V1, the mEC and the cornu ammonis regions (CA1, CA2, and CA3) of the dorsal hippocampus. We found that both the level of expression and laminar distribution of PNNs differed greatly between these areas (Fig. 1*A*,*B*). In the rat, PNNs were distributed across cell Layers in V1, apart from Layer I which is largely void of cell bodies, and the upper part of Layer V. The highest expression was found in Layers IV and V (Fig. 1*B*,*C*, left). In contrast, the superficial layers of mEC showed dense WFA labeling, while few PNNs were located in the deeper layers. The total fraction of PNN enwrapped neurons was, however, similar between V1 and mEC (8.4% in V1 and 7.9% in mEC, 5997 neurons counted in total).

The PNN expression of the dorsal hippocampus stands out with a very sparse and area-specific expression pattern (Fig. 1*A*,*C*, right). Only a few weak PNNs were found in CA1 and they were located in the stratum pyramidale, and some in stratum oriens. In CA2, however, the WFA staining was intense, enwrapping all cell bodies in the stratum pyramidale. The CA2 showed the highest density of WFA-enwrapped cells of all areas investigated, but the microstructure of the PNNs were more diffuse compared with the cortex (Fig. 1*B*,*D*). While a similar diffuse staining was observed in CA3 and in the granular cell layer of the DG, the intensity was much lower than in the CA2. In V1, the PNNs enwrapped the cell soma and a short section of the proximal dendrites. In contrast, PNNs in the mEC were similarly present around the cell soma but continued along long stretches of the dendritic segments (Fig. 1*D*, left and middle panel). In the CA2 of the hippocampus, on the other hand, PNNs located in the neuropil of the pyramidal cell layer enwrapped all cell somas but only a minor part of the processes (Fig. 1*D*, right).

**Figure 2.**
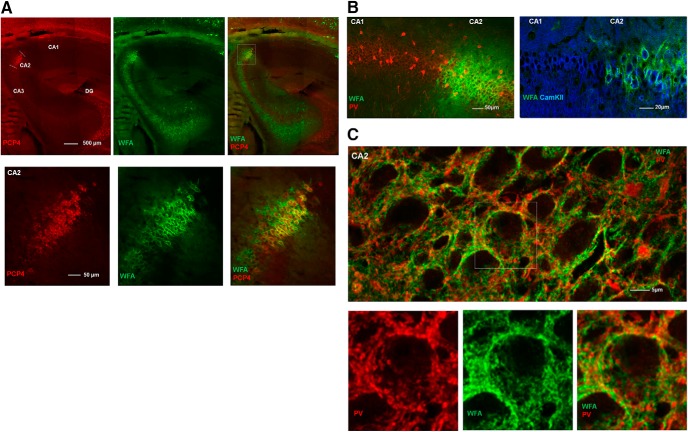
The hippocampal area CA2 shows dense expression of PNNs. ***A***, Sagittal section of a rat brain stained for PNNs (green) and the CA2-specific marker PCP4 (red) show complete overlap, indicating that the dense PNN-structures are selectively expressed in CA2. ***B***, The PNN-like ECM structures in CA2 of rat hippocampus primarily colocalize with CamKII-expressing, excitatory neurons and not PV+ neurons. Note the sharp border in WFA labeling between CA1 and CA2. ***C***, Maximum intensity projection from a z-stack in CA2. While the PNNs in CA2 are mostly associated with CamKII-expressing neurons, they also embed PV+ synaptic boutons surrounding CamKII-expressing principal neurons in the pyramidal cell layer.

To investigate whether the dense labeling in the dorsal hippocampus was limited to the CA2 region, we stained sections with the neuronal marker PCP4, which selectively label excitatory neurons in CA2 of the hippocampus (Botcher et al., 2014; Kohara et al., 2014). By counterstaining sections with WFA, we found that these markers overlapped, supporting that the PNN-positive neurons in CA2 differ from those in other sub-regions of the hippocampus (Fig. 2*A*). As the PNNs in CA2 were structurally different from the cortical areas, we went on to investigate if they also differed in cell-type specificity. Sections were stained for PV and CamKII, the latter labeling putative excitatory neurons. In CA2, we found that the large majority (∼80%) of neurons enwrapped in PNNs expressed CamKII (Fig. 2*B*). The PNNs in CA2 also enwrapped PV-expressing synaptic boutons onto the cell somata (Fig. 2*C*, lower image).

**Figure 3.**
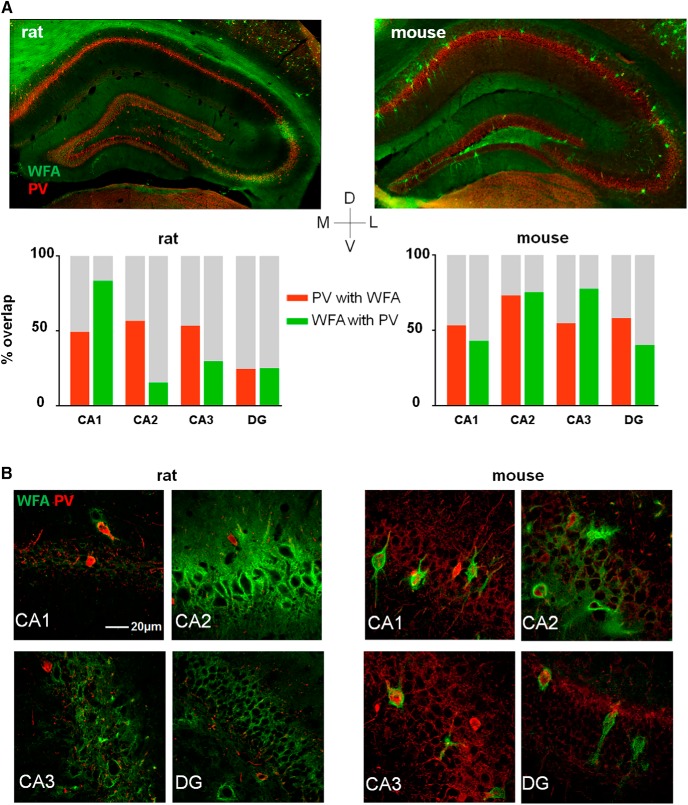
Expression of PNNs in the dorsal hippocampus varies between rats and mice. ***A***, ***B***, Coronal sections from a rat (left) and a mouse(right) brain stained with WFA to label PNNs (green) and parvalbumin (PV+; red). In the rat, almost no PNNs are expressed in the dorsal hippocampus with the exception of CA2, and part of the CA3, which is densely stained with diffuse PNN structures. In mouse, all subfields of the dorsal hippocampus show expression of large, structured PNNs. The overlap between PNN and WFA was calculated from counting identified PNNs and cell bodies in three sections from three animals (bar chart).

Overall, the special morphology and low density of PNNs in the hippocampus diverged profoundly from the other brain areas; hence we wanted to investigate if this was specific to the rat. While PNNs have been described in a range of species from birds to humans (Adams et al., 2001; Balmer et al., 2009), it remains unknown how the distribution and cell-type specificity varies between the widely used laboratory models mice and rats. The two species have nonetheless been used interchangeably in functional studies of PNNs. Indeed, comparing the distribution of PNNs in the rat and mouse hippocampi revealed large differences. In the mouse, PNNs were present in all sub regions of the hippocampus, with a structure similar to those in the cortical areas (Fig. 3*A*,*B*, right panels). In the rat hippocampus, however, we found very few discernable PNNs apart from that of the CA2 and to some extent in the CA3, which both showed a dense but diffuse matrix (Fig. 3*A*,*B*, left panels). Furthermore, while a large fraction of PNNs in mouse hippocampus colocalized with PV+ neurons, this was not the case in rats, with the exception of the few weak PNNs in the CA1 (Fig. 3*B*; Table 2). <20% of PNNs in CA2 in rats colocalized with PV+ neurons. The CA2 of mice was similar in terms of PNN structure, but not as densely packed as in the rat, and as much as 75% of PNNs enwrapped PV+ cells (Fig. 3*B*; Table 2).

**Table 2:** Colocalization between parvalbumin and WFA-positive PNNs in hippocampal areas of mice and rats

% PV overlapping with WFA			
Area	Rat	Mouse	*p* value
CA 1	49.6 ± 7.7	53.5 ± 4.7	0.34
CA 2	56.8 ± 3.5	73.4 ± 4.5	0.01
CA 3	51.3 ± 2.9	54.9 ± 4.1	0.53
DG	24.8 ± 6.8	58.2 ± 7.1	0.01
% WFA overlapping with PV			
Area	Rat	Mouse	*p* value
CA 1	83.6 ± 3.2	43.3 ± 4.8	<0.001
CA 2	15.7 ± 2.1	75.4 ± 5.9	<0.001
CA 3	29.3 ± 1.5	77.9 ± 6.6	0.002
DG	25.3 ± 5.8	40.5 ± 6.6	0.14

**Figure 4.**
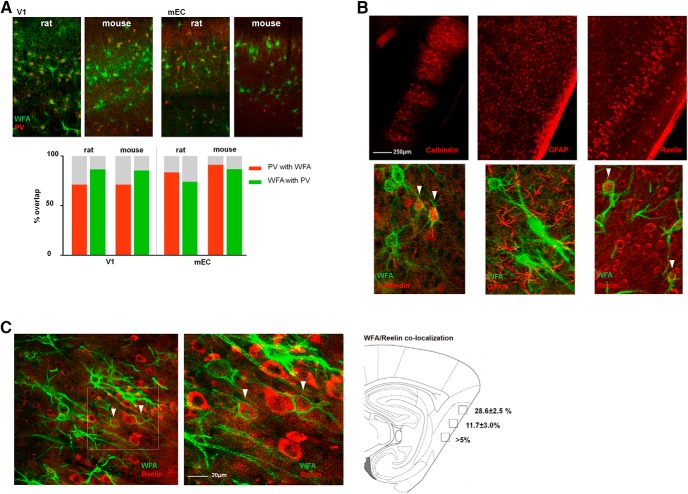
Colocalization of PNNs and cell type-specific markers show species and area-specific differences. ***A***, A majority of PNNs (WFA, green) colocalize with PV+ neurons (parvalbumin, red) in both V1 and mEC (left and right panels, respectively), but the overlap in mEC is different between mice and rats. The overlap (%) was calculated from counting identified PNNs and cell bodies in three sections from three animals (bar chart). ***B***, Sagittal sections from rat brain costained for PNNs and either calbindin (left), the astrocyte marker GFAP (middle), and reelin (right). Calbindin and reelin showed overlap with PNNs while no overlap was seen with GFAP. ***C***, A substantial part (28.6 ± 2.5%) of PNNs in the most dorsal part of mEC colocalizes with reelin-expressing cells.

**Figure 5.**
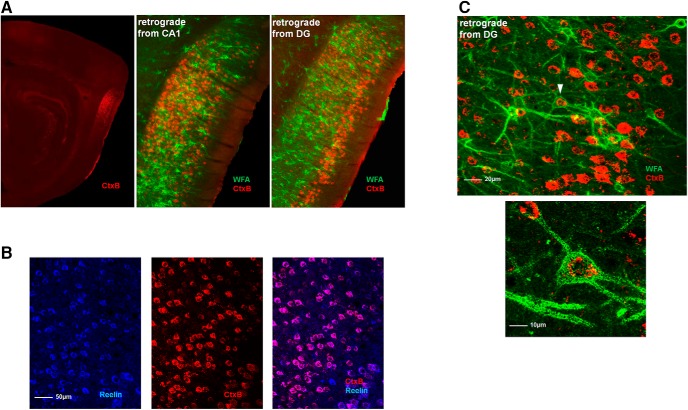
Retrograde tracing reveal projecting neurons from mEC to hippocampus. ***A***, We injected Cholera toxin subunit B (CtxB) into different areas of the dorsal hippocampus. Sagittal sections from a rat brain show PNNs (green) and cell soma of neurons in the mEC (red) filled with the retrograde tracer indicating that they project to dorsal hippocampus. ***B***, All neurons labeled by the tracer in mEC were reelin-expressing cells (blue), but not all reelin-expressing cells were labeled by the tracer. ***C***, Some neurons labeled by the tracer were enwrapped by PNNs, but a lower fraction than reelin cells.

The species comparison of the identity of PNN-positive cells was also conducted in the V1 and the mEC. In the V1, there was an almost exclusive colocalization between PNNs and PV+ in both species (Fig. 4*A*, left panel). In mEC, however, we found significant species differences, both in the fraction of PNNs colocalizing with PV+ cells and also the fraction of PV+ cells without PNNs (Fig. 4*A*, right panel). More PNNs colocalized with PV+ cells in mice (87%) compared with rats (74%). In rats, ∼25% of the PNNs in mEC colocalized with other cell types (Fig. 4*A*; Table 3). The mEC contains neurons with common morphologic characteristics but different phenotypes such as the spatially tuned grid-, border-, and head direction cells, and nonspatial cells (Sargolini et al., 2006; Kroppf et al., 2015; Ebbesen et al., 2016). The grid cells are found both among reelin-positive stellate cells that project to the DG, as well as the calbindin-positive pyramidal neurons (Kitamura et al., 2014; Tang et al., 2014; Sun et al., 2015). In an attempt to reveal the identity of the PNN-positive (non-PV) neurons and examine if some of them are potential grid cells, we stained brain sections from rats with several markers, including calbindin and reelin. In the most dorsomedial part of mEC we found considerable overlap between PNNs and reelin-positive cells (28% of PNNs colocalized with reelin; Fig. 4*B*,*C*) while only a small fraction (∼5%) of calbindin-expressing cells colocalized with PNNs (Fig. 4*B*). To test whether the reelin-positive neurons with PNNs were projecting to the hippocampus, we injected a retrograde tracer in CA1 and DG of dorsal hippocampus respectively, and stained sections with WFA. As expected (Witter, 2007) when injecting the tracer in DG, we found projecting neurons strongly labeled with the tracer in Layer II of mEC, and some labeling throughout Layer III (Fig. 5*A*). Counter-staining with reelin revealed an almost complete overlap between the tracer and reelin in mEC (Fig. 5*B*). The sections from the tracer experiments were also stained with WFA, revealing overlapping cells with the tracer and WFA, in particular in Layer II (Fig. 5*C*). However, only few such colocalized cells were found, indicating that most of the reelin-expressing WFA-positive cells are intrinsic to the entorhinal network, and only a subpopulation project to the dorsal hippocampus.

**Table 3:** Colocalization between parvalbumin and WFA-positive PNNs in mEC and V1 of mice and rats

% PV overlapping with WFA			
	Rat	Mouse	*p* value
V1	71.5 ± 2.4	71.6 ± 1.4	0.9
mEC	83.7 ± 1.4	91.5 ± 0.9	<0.001
% WFA overlapping with PV			
	Rat	Mouse	*p* value
V1	86.7 ± 2.4	85.6 ± 1.9	0.7
mEC	74.2 ± 2.8	87.1 ± 0.9	<0.001

**Figure 6.**
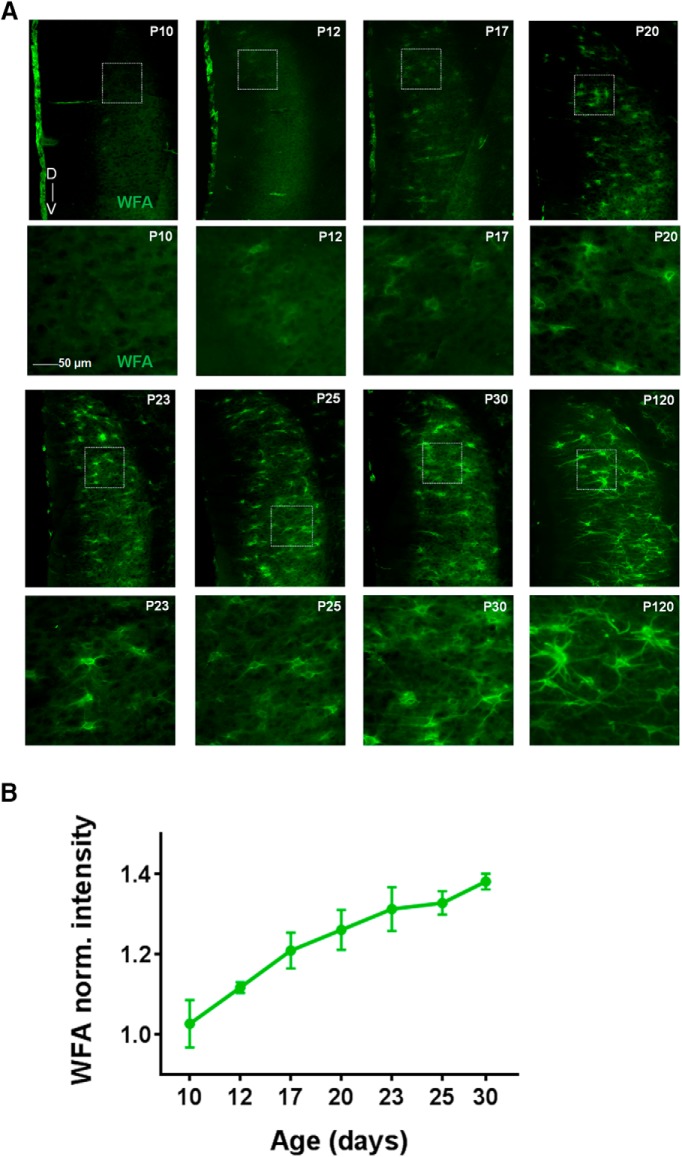
Development of WFA-positive PNNs in mEC of rats. ***A***, Sagittal sections from a rat brain stained for PNNs (WFA, green) at eight different time points during development. Insets are outlined under each time point. The first PNN-like structures appear around P12, and continue to condense throughout late postnatal development; first around the cell soma and later along the proximal dendrites. At P30, PNNs have developed an adult-like structure. ***B***, WFA intensity gradually increases during development. Fluorescence intensity across Layer II/III was measured for each time point at three different dorsoventral positions and normalized to background intensity in Layer I.

**Figure 7.**
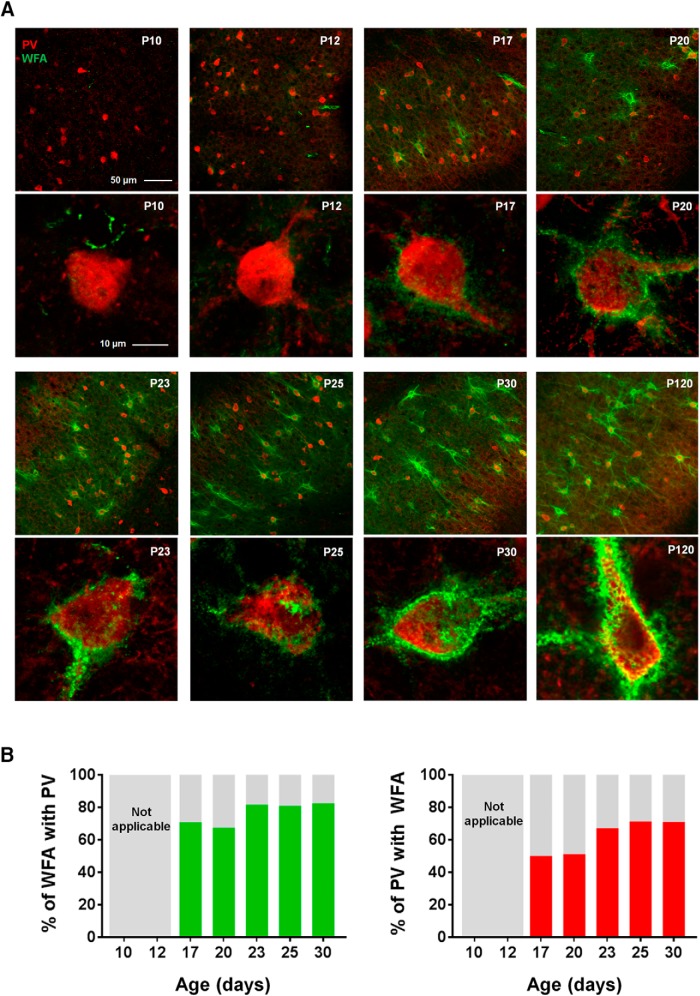
Development of PV+ cells and overlap with WFA-positive PNNs in mEC. ***A***, PV cells in mEC are present at P10, while the first clearly discernable PNNs appear at P17. ***B***, The colocalization between PV and PNNs increases gradually from the first point of measurement at P10 and stabilizes from P23, consistent with the development of PNNs.

In the cortex, the assembly of PNNs coincides with the closure of the critical period and may be linked to stabilization of functional phenotypes whose development is experience-dependent, such as ocular dominance and binocular matching in V1 (Pizzorusso et al., 2002; Wang et al., 2010). In the mEC, some head-direction cells show spatial tuning from early age, whereas the characteristic grid cell activity pattern appear much later (Langston et al., 2010; Wills et al., 2010; Bjerknes et al., 2014), suggesting experience-dependent development of grid cells similar to that of the sensory cortices. If the PNN assembly in the mEC follow a similar postnatal development as reported for V1 (Ye and Miao, 2013) remains unknown. To explore this we followed the PNN formation in mEC in postnatal development and found that the development of PNNs was strikingly similar to what has been observed in V1 with diffuse WFA-positive PNNs starting to appear around P12 but not fully developed until P30 (Fig. 6*A*). The most notable difference occurred between P17 and P20 when the PNNs became more clearly defined, after which we observed a gradual increase in WFA intensity and PNNs forming along longer stretches of the dendrites (Fig. 6*B*). We also costained sections with PV and quantified the overlap at different stages of development. In contrast to what has been reported from other cortical areas (Alcántara et al., 1993; Sugiyama et al., 2008), PV cells in mEC were present from the first time point (P10) we investigated (Fig. 7*A*, upper left panel). Due to the diffuse and weak appearance of the immature PNNs at the early stages an objective quantification of overlap between PNNs and PV+ neurons were not expedient (Fig. 7*B*). From P17 the overlap increased in a similar timeframe to PNN development.

**Figure 8.**
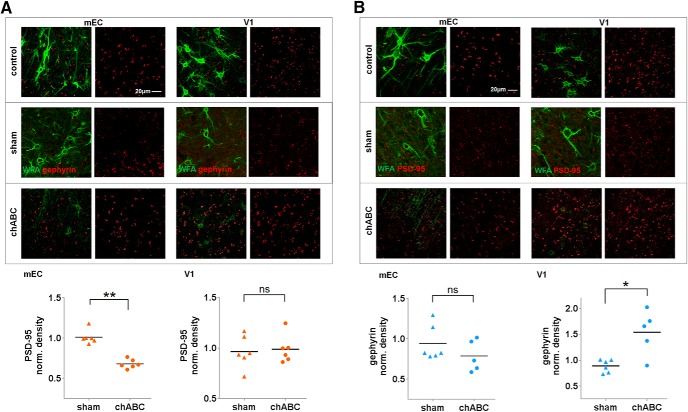
Local injections of the enzyme chABC into the rat brain disintegrate the PNNs and affect the number of PSD-95 and gephyrin puncta. ***A***, Sagittal sections of the brain from a rat treated unilaterally with chABC (bottom row) or aCSF (sham, middle row) and control hemisphere (top row), and stained for PNNs (green) and gephyrin puncta (red) in V1 and mEC. Quantification of puncta was conducted by using the spot detection algorithm in Imaris, and performed on two to three sections from three rats injected with chABC and three rats injected with aCSF (***A***, ***B***, lower panels). In mEC, there were no significant changes in the number of gephyrin puncta (*p* = 0.25, Mann–Whitney *U* test), but an increase in V1 (*p* = 0.03, Mann–Whitney *U* test). ***B***, The chABC treatment caused a significant reduction of PSD-95 puncta (red) in mEC (*p* = 0.002, Mann–Whitney *U* test), and no significant effect in V1 (*p* = 0.7, Student’s *t* test). Data were normalized to the contralateral control hemisphere for each animal used, line indicates mean values.

Several lines of evidence point to a role of PNNs in stabilizing synaptic connections between neurons (Dityatev et al., 2010; de Vivo et al., 2013). Despite the profound increase in plasticity after enzymatic degradation of PNNs in the adult brain, it remains unclear how removal of PNNs affects the stability and organization of synapses. To investigate this, we used the enzyme chABC to degrade PNNs. Adult rats were injected unilaterally with chABC or aCSF (sham treatment) in V1 and mEC, and euthanized after 7 d. Brain sections were stained with WFA in combination with the postsynaptic markers PSD-95 or gephyrin, labeling putative excitatory and inhibitory synapses, respectively. The effects of PNN removal showed large differences between brain areas. While removal of the PNNs from the mEC caused a reduction in the number of PSD-95-expressing puncta (*p* < 0.002, Mann–Whitney *U* test; Fig. 8*B*, left panels), the effect of chABC treatment in V1 was an increase in the number of gephyrin puncta (*p* < 0.03, Mann–Whitney *U* test; Fig. 8*A*,*B*, right panels). Sham-treated animals were not different from controls for any marker in any of the areas examined.

**Figure 9.**
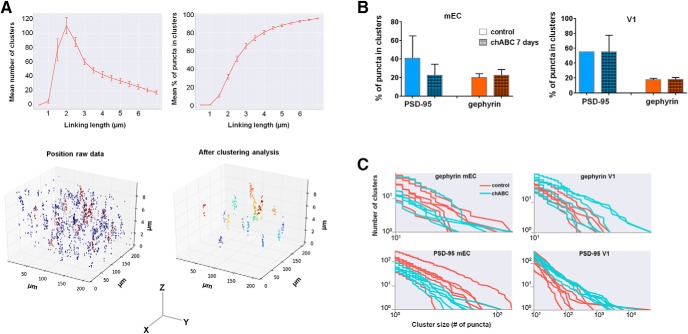
Distribution and clustering of PSD-95 and gephyrin puncta in mEC and V1 after chABC treatment. ***A***, Overview of the clustering method and differences in clustering as a result of different linking length. Lower left panel shows the raw data of identified gephyrin puncta in mEC from a z-stack, visualized in 3D space. The nonclustered puncta are shown in blue, while clusters are shown in red. Lower right side shows the identified clusters, when using 2 µm as linking length. Note the difference in axis range between *z* and *x y*, causing the elongated shape of the clusters. ***B***, The percentage of puncta within clusters in controls and chABC-treated rats in mEC and V1. We did not detect significant differences between the groups. There was a tendency toward reduced clustering after chABC treatment in mEC, but overall, high variation between the data sets, in particular for excitatory puncta (PSD-95). ***C***, Examples of distribution of cluster sizes, from six measurements (z-stacks) in mEC and V1.

Given that chABC treatment caused dramatic changes to the number and inhibitory-excitatory distribution of puncta, we wanted to investigate whether the organization was affected. We used the positional data of the different synaptic markers and an adapted version of the FOF algorithm to cluster the data (Fig. 9*A*). The fraction of puncta located in clusters after treatment with chABC were calculated for each synaptic marker in both areas and compared with controls. Although PSD-95 puncta in mEC showed a tendency to be less clustered after chABC treatment, we found no significant changes in synaptic organization for either of the two brain areas (Fig. 9*B*,*C*).

## Discussion

We have shown that the expression, microstructure, and cell specificity of PNNs is highly variable between three brain areas that differ in connectivity, architecture and cognitive functions. Our data show that the general assumption that PNNs primarily colocalize with PV+ neurons is not evident for all brain areas or across different species. In particular, while PNNs mainly colocalize with PV+ neurons in visual cortex, they predominantly colocalize with CaMKII and PCP4-expressing neurons in CA2 of the hippocampus. In the grid cell network of the mEC, PNNs embody several cell types, predominantly PV- and reelin-expressing cells. Moreover, the differences between PNN expression in rats and mice emphasize the importance of taking into account species differences when dissecting the functional roles of PNNs.

The PNNs are believed to serve several functions in the brain, from ion buffering to providing a rigid structure to maintain synaptic integrity and organization (Frischknecht et al., 2009; Beurdeley et al., 2012; Cabungcal et al., 2013; Dick et al., 2013; Tsien, 2013; Morawski et al., 2015). In more general terms, PNNs seem to have an important function in restricting adult brain plasticity. In line with this, removal of PNNs, both by enzymatic degradation and by partial genetic knock-out, has been shown to increase plasticity comparable to juvenile levels in several areas of the brain (Pizzorusso et al., 2002; Gogolla et al., 2009; Carulli et al., 2010; Happel et al., 2014; Slaker et al., 2015; Lensjø et al., 2017).

### PNN expression and plasticity

In line with the functional differences between the three areas investigated, we found large differences in the expression of PNNs (Fig. 1). The PNNs in V1 had a uniform distribution with a structure usually described packing around the soma and proximal dendrite (Wegner et al., 2003). In the mEC, however, PNN expression was limited to Layers II and III. The nets in mEC also packed around the soma but extended along long stretches of the dendrites. This difference in distribution pattern may reflect layer-specific functional roles of neurons in the mEC with, e.g., grid cells being most abundant in Layers II and III. Approximately 10% of all the neurons in V1 and mEC of rats were enwrapped in a PNN. This is comparable with previous work in macaque, albeit somewhat higher (5% in V1; Mueller et al., 2016). In contrast, the dorsal hippocampal areas CA1 and CA3 have very low expression of PNNs, which may be indicative of the high level of plasticity in these areas. Local ensembles of hippocampal place cells can take part in different cell assemblies in an unpredictable manner (Leutgeb et al., 2004). This stands in contrast to local ensembles of entorhinal grid cells which show a rigid spatial and relationship with coherent realignment during remapping (Fyhn et al., 2007; Stensola et al., 2012). Further work should clarify if the lack of PNNs in CA1 and CA3 support a flexible population code. Moreover, a potential role of PNNs for stabilizing the grid cell network needs further investigation.

The hippocampus maintains a high degree of plasticity throughout life. This is documented through easy induction and strong long-term potentiation of synapses, NMDA receptor-dependent plasticity of many hippocampus-dependent cognitive processes and the ability of cell ensembles to independently reorganize and change in response to small changes in external inputs (remapping). Within the hippocampus we found large variation in the expression of PNNs. The low expression of PNNs in the CA1 region of the dorsal hippocampus contrasted with the dense expression of PNN-like structures in the CA2 region. Interestingly, the CA2 area is characterized by low levels of plasticity compared with the surrounding areas (Dudek et al., 2016).

Taken together, it is tempting to speculate that there is a relationship between PNN expression and the level of plasticity, where neural networks with lower levels of plasticity correlate with abundant expression of PNNs while the opposite seem to be true for regions with high degree of plasticity such as the CA1 region of the dorsal hippocampus. However, it should be noted that PNNs only surround a small fraction of the neurons, suggesting that the increased plasticity observed after enzymatic degradation of PNNs is likely to be network effects including more than the neurons surrounded by PNNs.

### PNNs in hippocampus of rats and mice

In accordance with previous work in mice (Yamada and Jinno, 2013, 2016), we found sparse but clearly defined PNNs across most areas of mouse dorsal hippocampus, in particular in CA1. This was in contrast to the rat CA1, where only few and weakly labeled PNNs were discernable (Fig. 3). The species difference was also prominent for the colocalization between PNNs and PV-expressing neurons. Among the few PNNs found in the rat hippocampus, only a relatively small fraction were colocalized with PV+ neurons, while a high fraction colocalized in most areas of mouse hippocampus. It remains an open question if these differences have any functional consequences for network computations in the two species.

The dense expression of PNNs with a diffuse microstructure in the CA2 region of the hippocampus was remarkably different from PNNs in CA1 and neocortex. The PNN-like structures in CA2 of rats did not primarily colocalize with PV+ cells but rather with pyramidal neurons expressing CamKII, indicative of excitatory neurons. The complete overlap in colabeling of WFA and the CA2 neuron-specific marker PCP4 confirmed that the expression of this dense matrix was selective to CA2, which is in accordance with recent work (Carstens et al., 2016). These observations add to a growing body of evidence indicating that the CA2 is a functionally and structurally distinct region of the hippocampus with unique features (Jones and McHugh, 2011; Dudek et al., 2016). The principal neurons found in this area differ from the areas around in terms of morphology, gene expression, physiologic properties, and connections to other areas (Caruana et al., 2012; Hitti and Siegelbaum, 2014; Mankin et al., 2015), and CA2 is more resistant to ischemia-induced cell death (Sadowski et al., 1999; Zao et al., 2007). The CA2 has proven highly resistant to experimentally induced long-term potentiation (at Schaffer collateral synapses) compared with CA1 (Zao et al., 2007). Recent work shows that enzymatic removal of PNNs in CA2 of juvenile mice (P14-18) increases plasticity in this area, to a level comparable to CA1 (Carstens et al., 2016). Similar to chABC treatment, caffeine has also been shown to induce potentiation of synaptic responses in CA2 through its effect of adenosine receptors which are highly expressed in this area (Simons et al., 2011). Thus, it appears that while the CA2 is an area resistant to synaptic change under normal conditions, it may be that a state switch such as that induced by PNN removal or caffeine opens for synaptic plasticity in the circuitry. Taken together, this suggest a highly important functional role for PNNs in the CA2, and that while the PNNs of the CA2 are structurally different to other areas, they likely serve many of the same functions.

Several studies have been using the hippocampus as a model area for investigations of PNN function (e.g. Hylin et al., 2013). However, the remarkable low expression of PNNs in the hippocampus of both mice and rats argues against using the hippocampus as a model to understand the role of PNNs. despite this recent work claims that the effects of chABC treatment in dorsal hippocampus on behavioral performance and gene expression were due to removal of the PNNs (Donato et al., 2013; Hylin et al., 2013; Yamada et al., 2015). While the effects observed were statistically significant, they may be attributed to chondroitin sulfate proteoglycans in general, rather than the few PNNs present in the area. However, for cortical areas such as visual cortex and the perirhinal cortex, the plasticity effects of chABC treatment have been verified by genetic deletion of the link protein, Crtl-1 (Carulli et al., 2010; Romberg et al., 2013), which is a key component and specific for the PNNs. It may be that it is the lack of PNNs that allows for the hippocampus to retain the unique plasticity necessary for learning and memory processing throughout life.

### PNNs in V1

The V1 is the brain area where the function of PNNs has been most investigated. Here, PNNs have been shown to assemble in parallel with the maturation of the inhibitory network of PV+ neurons as the critical period for ocular dominance plasticity ends (Pizzorusso et al., 2002; 2006; Liu et al., 2013; Ye and Miao, 2013). Perturbations of PNNs in V1 by chABC or deletion of the link protein Crtl-1 reopens for ocular dominance plasticity in V1 (Pizzorusso et al., 2002; Carulli et al., 2010). We found strong expression of PNNs in V1 across all cell layers, except for Layer I (Fig. 1), and high degree of colocalization with PV in both mice and rats, with values very similar to what has previously been reported (Fig. 4*A*; Beurdeley et al., 2012; Liu et al., 2013; Ye and Miao, 2013). While the adult V1 network shows limited degree of experience-dependent plasticity in adulthood, prolonged sensory deprivation does cause a shift in ocular dominance (Sawtell et al., 2003; Tagawa et al., 2005; Sato and Stryker, 2008). This process differs from critical period plasticity in that it is largely dependent on other cellular mechanisms, and on the action of matrix metalloproteinases (MMPs) (Ranson et al., 2012; Pielecka-Fortuna et al., 2015). These enzymes are activated on changes in activity and degrade ECM molecules locally. Blocking the action of MMPs abolishes the effect of sensory deprivation in adult animals (Pielecka-Fortuna et al., 2015), suggesting that PNN modification is an important part of the process.

### PNNs in mEC

PNNs have been reported in the mEC of humans (Pantazopoulos et al., 2010; Lendvai et al., 2013). To our knowledge, the current study is the first to quantify PNNs in mEC of rodents. The dense expression of PNNs in Layers II and III and high colocalization with PV+ cells that are known to be a key player in the grid cell network, indicates a role for the PNNs in the circuitry for spatial navigation (Fig. 4). The distribution of PNNs corresponds to the PV expression pattern (Wouterlood et al., 1995; Fujimaru and Kosaka, 1996) and to the distribution of PNNs in human mEC where the expression is also mainly located to Layers II and III (Lendvai et al., 2013). The mEC has been proposed to be a hub in a distributed network for navigation in the brain. A subpopulation of neurons, the grid cells, shows a remarkable activity pattern of repetitive firing fields spanning the course of the environment visited by the animal (Hafting et al., 2005). The grid map is stable over time and ensembles of grid cells operate in synchrony and retain its temporal relationship indicating a hard-wired neural network. The grid cells are likely interconnected through a network of mono-synaptically coupled PV+ cells, which is believed to contribute to the rigid activity pattern of the grid cell network (Couey et al., 2013, Buetfering et al., 2014). The large overlap between PV+ cells and PNNs described here and the plasticity limiting role of PNNs in other cortical areas, suggests that the PNNs could play a role in grid cell stability. Emerging evidence suggests that the grid cells are likely to be both stellate (reelin-positive) and pyramidal (calbindin-positive) cells (Sun et al., 2015), both of which partly colocalized with PNNs. While the overlap with calbindin was small, we found a substantial part of the PNNs colocalizing with reelin-expressing cells (Fig. 4*B*,*C*). Based on our tracer experiments it appears as if there are different populations of stellate cells, with a subset of hippocampus projecting stellate cells that colocalized with WFA (Fig. 4*C*). While this could also be an effect of the efficiency of the tracer injection, it seems likely that most of the WFA-expressing stellate cells do not project to CA1. It remains to be investigated if the PNN expression of these subgroups is associated with functional properties.

While the underlying mechanisms remain mostly unknown, the occurrence in time of stable spatial representations of the grid cells in mEC (Langston et al., 2010; Wills et al., 2010; Bjerknes et al., 2014) coincide with the closure of critical period plasticity in other cortical areas. We show that the maturation of PNNs in this region closely follows the timing reported for development of grid cells, and is very similar to the PNN maturation seen in primary sensory cortices (Ye and Miao, 2013; Fig. 6). However, unlike in other cortical areas (Alcántara et al., 1993), we found that PV+ neurons in mEC were present already at P10 (Fig. 7*A*). This might appear contradictive to the notion that PV+ neurons and PNNs mature together, but recent work investigating the development of the neuronal network in mEC has demonstrated that PV+ cells show adult phenotypes from P14-P20 (Donato et al., 2017), i.e., during the same period that the colocalization between PV+ cells and PNNs increases dramatically and stable spatial representations appear (Fig. 7*B*). Hence, our data suggests that PNNs may contribute to secure a rigid neural network which may be a prerequisite for the stable spatial representations of grid cells in adulthood. This also suggests that PV itself is not a reliable marker for activity and cellular maturation. Whereas functional investigations of mEC after PNN manipulation are needed to reveal their role in spatial navigation, their dense expression in mEC and the cell types being enwrapped in PNNs point toward a role in stabilizing the network.

### Synaptic stability and distribution

To study whether PNNs affect synaptic stability we used the bacterial enzyme chABC to degrade chondroitin sulfate glycosaminoglycans unilaterally in V1 and mEC. This treatment effectively collapses the PNNs and abolishes WFA staining (Brückner et al., 1998). Previous work has shown that chABC treatment causes changes to the number of synapses and spine motility in hippocampus and V1, respectively (de Vivo et al., 2013; Donato et al., 2013). We found large effects on the number of putative inhibitory and excitatory puncta, and the ratio between these after chABC treatment. However there was large variability between brain areas (Fig. 8). In mEC, there was a reduction in the number of identified PSD-95 puncta, while V1 showed a shift toward more gephyrin puncta. These changes may be indicative of a network in a high plasticity state, as similar effects have been observed after a period of environmental enrichment, a paradigm known to cause increased plasticity (Donato et al., 2013). The different effects of PNN removal on puncta between V1 and mEC may point to different functional effects of the treatment in the two regions. This may be explained by differences in the neural architecture of the networks and composition of sub-populations of neurons. It may be that removal of the PNNs set the network in a plastic state where the plasticity changes that may occur are network specific. Whereas the effect of chABC on V1 is known, the functional effects of chABC treatment in mEC remain elusive and require further investigations. We interpret these results with some caution as the chondroitin sulfate proteoglycans embedded in PNNs only make up 2-5% of the total chondroitin sulfate proteoglycans in the rat brain. It is therefore likely that some of the effects we observe are due to degradation of ECM in general (Deepa et al., 2006). However, as the PNNs enwrap individual synapses onto the soma of neurons (Fig. 2; Mueller et al., 2016), their removal by itself could produce effects on synapse organization and integrity.

To analyze the distribution of puncta, we used an adapted version of the FOF algorithm to define clusters of puncta. The distribution of puncta may indicate if the treatment of chABC caused any changes to the distribution of synapses onto the neurons. To the best of our knowledge, this is a novel tool in the study of synaptic organization. Compared with other clustering algorithms, the FOF algorithm has the advantage of directly returning which puncta belong to a given cluster. Furthermore, it is purely geometric and does not impose any assumptions on the data in the calculations, and is reasonably fast with a time complexity of O = (*n* ^2^), where *n* = total number of puncta. This can be further improved by using an already existing algorithm that compares puncta on a grid, giving a time complexity of O = Nlog(n). This may be a highly useful tool for the study of structural network organization and stability.

We did not detect significant differences between the control and chABC-treated areas, but a tendency for PSD-95 puncta in mEC to be less clustered after chABC treatment (Fig. 9*B*). As a general note, however, there was large variability in the data between different animals, in particular for the PSD-95 puncta, and our analysis does not take into account the reduction in total number of puncta. additional experiments using repeated spine imaging are needed to understand the role of PNNs in organization and stability of the synaptic circuitry.

The different levels of expression, cell-type specificity and appearance of the PNNs between brain regions and species may indicate that the PNNs may have different functions depending of the network architecture and function. To reveal the function of the PNNs a systems neuroscience approach is needed combining structural mapping of their distribution and microstructure with functional investigations using targeted manipulations of the PNNs and direct assessment of effects on network function and structure.

## Synthesis

Reviewing Editor: Thomas McHugh, RIKEN Brain Science Institute Decisions are customarily a result of the Reviewing Editor and the peer reviewers coming together and discussing their recommendations until a consensus is reached. When revisions are invited, a fact-based synthesis statement explaining their decision and outlining what is needed to prepare a revision will be listed below. The following reviewer(s) agreed to reveal their identity: Rebecca Piskorowski, Barbara Sorg.

SYNTHESIS

The authors have made a number of key changes in the revised manuscript, including improving the introduction and discussion and adding additional data. While the reviewers appreciate these efforts, commenting, “The presentation and interpretation of the results in the manuscript are very much improved.” they continue to have serious reservations about the interpretability of the chABC data presented in figures 8 and 9.

REVIEWER COMMENTS

There are two major issues that both reviewers raised. First is the very small N (3 treated animals, 2 sham controls). One reviewer commented, “These numbers are very low for number of animals used. For all animals, the data were counted on the non-injected side. For all 5 animals, do these “control” measurements have the same distribution? Are the means different? Is there enormous variability? How well do the measurements of each animal represent the entire set? If the control data from one animal does in fact represent the data of the 5, then it should be possible to compare measurements of the manipulated sides of the brain. Also, for the manipulations, if there is limited variation between animals for the chABC injection and sham injection, i.e. if the data from one slice represents the population. Pooling data and using a t-test (normal distribution) or the Mann-Whitney (not normal) should be possible if all assumptions are valid...However, the authors failed to communicate that they ensured the proper assumptions were met for their data. An N of 2 is really not exemplary, and if data from slices are pooled for each animal, 3 is needed to determine if the distribution is normal. There also appears to be a lot of variability in the chABC data”, while the other added, “The addition of two sham animals is problematic, in that first, to do a statistical comparison, a minimum of three animals must be used. Second, it would appear from Figure 8 that staining in sham animals looks heavier from that of “Control” animals.”

The second issue raised relates to the continued use of the term synaptic puncta. Reviewer #2 notes, “The “synaptic puncta” term is still used throughout the manuscript. Puncta analysis cannot be done in the way it is shown to determine the number of puncta on WFA. The PNN is around the cell, which means the analysis of synaptic proteins would need to be done by analyzing the markers PV and CamKII, since this is actually where synaptic connections are formed rather than at the PNN itself. Looking at synaptic markers diffused within the PNNs does not give confidence that these markers are actually close (i.e., forming synapses with) the underlying cell. I am not sure what they are counting in Figure 8 to determine the number of puncta. (Methods indicates “all puncta that are connected to each other through a network of linked puncta”0, but there are no labels (arrows) to indicate what is meant by puncta in Figure 8. There may well be puncta here, but how is it differentiated from background staining?”

EDITORS SUGGESTION

Aside from the issues concerning the chABC experiment in Figures 8 and 9, the reviewers agree that although largely descriptive, the study is well conducted and reported and would support publication of a paper containing figure 1 thru 7.

As a result, one option I'd like to offer you as authors is to revise the manuscript without the chABC experiment and resubmit it to be assessed by the editor. If you are unwilling to remove these data then the final decision will reflect the reviewers continued concerns.

Minor issues:

1. Typos:

Line 381: “...While the effects observed were significance ...”

Line 487: “...and species indicate may indicate that the PNNs...”

2. The authors should use arrows to point out single- and double-labeled cells in the figures.
